# Biocompatibility and Surface Properties of TiO_2_ Thin Films Deposited by DC Magnetron Sputtering

**DOI:** 10.3390/ma7064105

**Published:** 2014-05-27

**Authors:** Francisco López-Huerta, Blanca Cervantes, Octavio González, Julián Hernández-Torres, Leandro García-González, Rosario Vega, Agustín L. Herrera-May, Enrique Soto

**Affiliations:** 1 Centro de Investigación en Micro y Nanotecnología, Universidad Veracruzana, Calzada Ruiz Cortines 455, 94294 Boca del Río, Veracruz, Mexico; E-Mails: blanca.cervantes@gmail.com (B.C.); julihernandez@uv.mx (J.H.-T.); leagarcia@uv.mx (L.G.-G.); leherrera@uv.mx (A.L.H.-M.); 2 Instituto de Fisiología, Benemérita Universidad Autónoma de Puebla, Av. San Claudio 6301, Col. San Manuel, 72570 Puebla, Mexico; E-Mails: octavio.gp.21@gmail.com (O.G.); axolotl_56@yahoo.com.mx (R.V.); esoto24@gmail.com (E.S.); 3 Licenciatura en Biomedicina, Benemérita Universidad Autónoma de Puebla, 13 Sur 2702, Col. Volcanes, 72410 Puebla, Mexico

**Keywords:** biocompatibility, dorsal root ganglion neurons, direct current magnetron sputtering, thin film, TiO_2_

## Abstract

We present the study of the biocompatibility and surface properties of titanium dioxide (TiO_2_) thin films deposited by direct current magnetron sputtering. These films are deposited on a quartz substrate at room temperature and annealed with different temperatures (100, 300, 500, 800 and 1100 °C). The biocompatibility of the TiO_2_ thin films is analyzed using primary cultures of dorsal root ganglion (DRG) of Wistar rats, whose neurons are incubated on the TiO_2_ thin films and on a control substrate during 18 to 24 h. These neurons are activated by electrical stimuli and its ionic currents and action potential activity recorded. Through X-ray diffraction (XRD), the surface of TiO_2_ thin films showed a good quality, homogeneity and roughness. The XRD results showed the anatase to rutile phase transition in TiO_2_ thin films at temperatures between 500 and 1100 °C. This phase had a grain size from 15 to 38 nm, which allowed a suitable structural and crystal phase stability of the TiO_2_ thin films for low and high temperature. The biocompatibility experiments of these films indicated that they were appropriated for culture of living neurons which displayed normal electrical behavior.

## 1. Introduction

Recent advances in the complementary metal oxide semiconductor (CMOS) and micro-electro-mechanical systems (MEMS) technologies have allowed the fabrication of CMOS-MEMS devices that can be used on diverse areas such as agriculture, communications, environment, medicine and biomedical applications [1,2,3,4,5]. The biocompatibility is the capability of not to elicit a negative physiological response while maintaining functionality within the body environment [6,7]. Materials need to be screened and verified for their biocompatibility before use in living organism according with international norms [8]. Many of the materials implemented in CMOS-MEMS devices are opaque or dark, which causes problems to examine their biocompatibility using traditional methods [9]. One solution is to use a 3-(4,5-dimethyl-2-thiazolyl)-2,5-diphenyl-tetrazolium bromide (MTT) assay or to develop systems based on transparent material such as indium-tin oxide (ITO) and titanium dioxide (TiO_2_) [10,11,12,13]. For instance, Titanium alloys are commonly employed to manufacture hard tissue replacement, including dental implants, bone plates and artificial hip joints [14,15,16,17,18]. These materials have positive characteristics such as good mechanical strength, lightweight, biocompatibility, and corrosion resistance, which generally depend of their surface properties [14,19].

TiO_2_ films are an option for use in solar cells, photocatalysis, sensors, photoelectrolysis, biomedical devices, and biomaterials [20,21,22,23,24,25]. TiO_2_ has three main polymorphic phases: rutile, anatase and brookite. Both anatase and rutile phases have tetragonal crystal structures but belong to different space groups. The low-density solid phases have less stability and undergo transition to rutile in the solid state. This phase transition is accelerated by heat treatment and can occur at temperatures between 450 and 1200 °C. It depends on the following parameters: initial particle size, initial phase, dopant concentration, reaction atmosphere and annealing temperature [26,27]. The TiO_2_ films can be synthesized by different methods such as sol-gel, thermal spraying and physical vapor deposition [11,28,29,30]. In addition, the direct current (DC) reactive magnetron sputtering method is used to control the composition and structure of TiO_2_ films, and it provides a strong substrate adhesion and large area with uniform thickness. This method can easily adjust the deposition conditions and offers a high deposition rate using argon gas, which is attractive for large-scale production. For biomedical applications, these films must have biocompatibility to avoid toxic effects on biological systems. Biocompatibility tests must evaluate the biomaterial cytotoxicity and the functionality of the biological system when it is exposed to this biomaterial [31,32,33,34,35,36,37]. Biocompatibility studies of an inorganic material require complex experiments both *in vitro* and *in vivo* in order to test the local and systemic effects of the material on cultured cells, tissue sections, and the whole body. *In vitro* cell culture tests are often used to screen the short term biocompatibility of inorganic material. These tests are sensitive, reliable, convenient, and reproducible screening methods. The study of the *in vitro* biocompatibility offers a controlled environment to test specific cellular and molecular processes. It has less variability than the *in vivo* tests and facilitates the experimental replication. In order to use TiO_2_ thin films in the biomedical microsensors fabrication, these films must satisfy the *in vitro* biocompatibility tests. Next, these microsensors must be satisfactory evaluated *in vivo* animals. Finally, they could be used in clinical studies. Thus, we present a biocompatibility analysis and surface characterization of TiO_2_ thin films deposited on a quartz substrate, which are generated by reactive DC magnetron sputtering and annealing at different temperatures.

## 2. Results and Discussion

### 2.1. Physical Properties of the TiO_2_ Films

**Figure 1:**
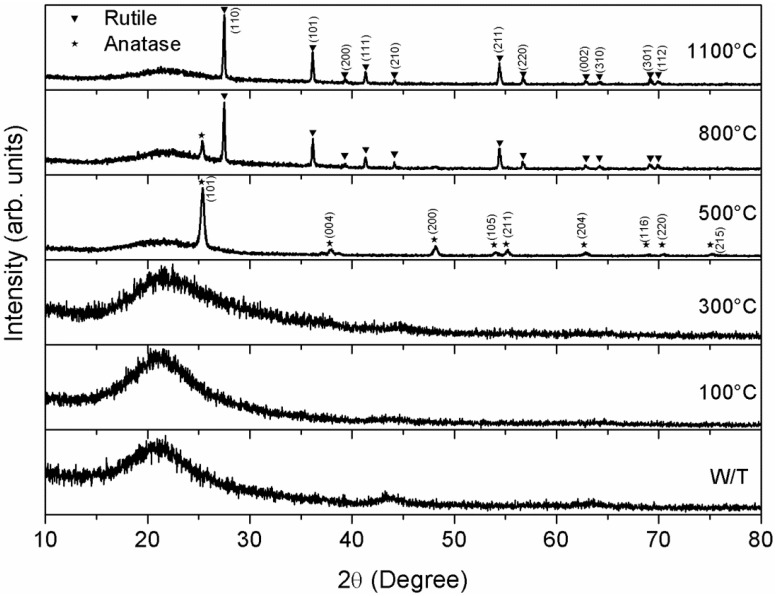
X-ray diffraction patterns for films annealed at different temperatures.

The TiO_2_ thin films are characterized using an X-ray diffractometer (XRD) at room temperature. The microstructural properties of the films were measurement using XRD (Bruker, D8 Advance, Karlsruhe, Germany) with CuKα radiation in the 2θ degree angle between 20° and 80°. Figure 1 shows the XRD data of TiO_2_ thin films annealed to temperatures between 100 and 1100 °C. The X-ray diffraction pattern of the TiO_2_ thin films shows a maximum signal about 20°, which is due to the quartz substrate. Similar X-ray diffraction patterns are obtained of TiO_2_ thin films annealed between 100 and 300 °C. However, X-ray diffraction patterns of TiO_2_ thin films annealed at 500 °C showed sharp diffraction peaks that can be assigned to the anatase TiO_2_ phase with tetragonal body-centered structure (ICDD: 00-021-1272) [38]. 

In addition, the X-ray diffraction pattern of the TiO_2_ thin film annealed at 800 °C showed the coexistence of anatase-rutile, in where the intensity of rutile phase over anatase phase increased due to increment of the thermal annealing treatment. The rutile TiO_2_ phase (ICDD: 01-089-4920) [39] showed a tetragonal body-centered structure and a crystallographic orientation in plane (110). Finally, TiO_2_ thin film annealed at 1100 °C had a set of sharp diffraction lines of the rutile TiO_2_ phase. We conclude that the films have a strong orientation in the direction normal to the substrate surface.

The average crystalline grain size of anatase and rutile phase of TiO_2_ thin films are shown in Table 1. The increment of the thermal annealing treatment caused that the grain size of the anatase and rutile TiO_2_ phase had an increased and decreased, respectively. The crystalline grain size is obtained by the X-ray line profile analysis through of the Warren-Averbach method using a Pearson VII function (McKeehan) [40].

**Table 1:** Average values of the crystalline grain size of anatase and rutile phase TiO_2_ films annealed at different temperatures.

Heat Treatment (°C)	Crystalline Size (nm)	
	Anatase TiO_2_	Rutile TiO_2_
500	15.9	–
800	20.8	37.5
1100	–	32.7

### 2.2. Biocompatibility Test

**Figure 2:**
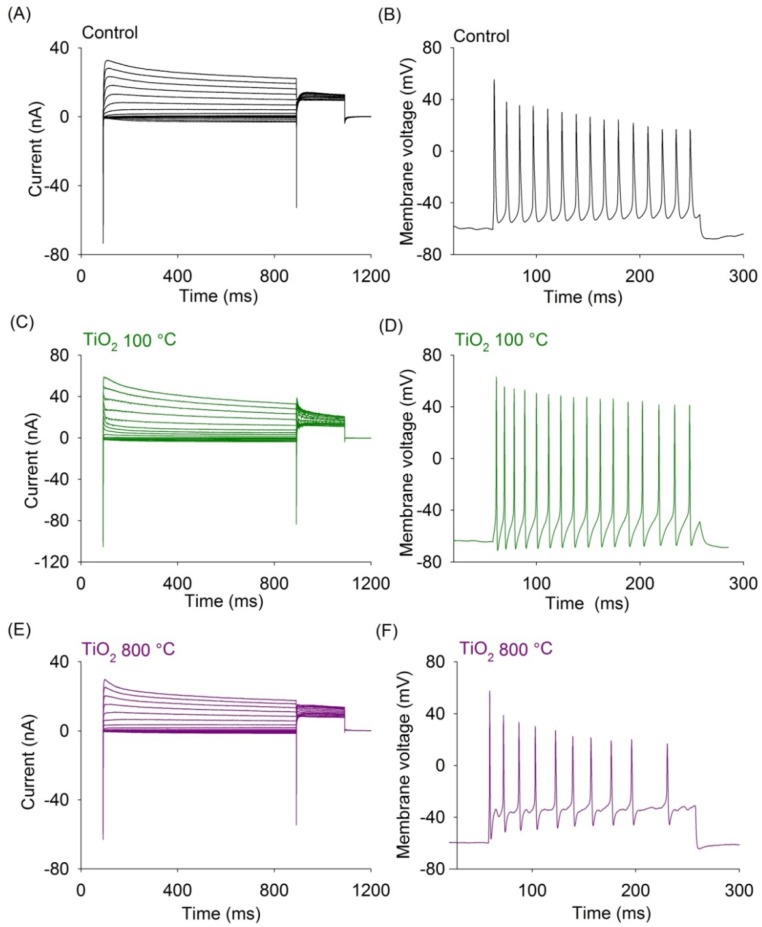
Representative membrane ionic currents and membrane voltage response of DRG neurons cultured in control substrate and TiO_2_ thin films surfaces. (**A**,**C**,**E**) ionic currents generated with an 800 ms pulse (from −110 to +40 mV) followed by 200 ms test pulses to 0 mV (*V*_H_ = −60 mV). Neurons grown on control glass surface and those grown on TiO_2_ films annealed at 100 and 800 °C showed similar set of currents with an initial rapid inward current followed by a slowly inactivating outward current; (**B**,**D**,**F**) representative voltage responses to current pulse injection (100, 400 and 300 pA, and *V*_m_ = −60 mV) of DRG neurons cultured in control substrate and TiO_2_ thin films annealed at 100 and 800 °C. Recordings of the voltage response of neurons to a current pulse showed typical action potential discharge showing the functionality of the neurons growth in control and TiO_2_ films.

The control glass substrate and TiO_2_ thin film surfaces of the neurons displayed typical membrane ionic currents activated by voltage variations, including a fast inward current at the beginning of voltage clamp pulse (Na^+^ current) and ensuing outward currents (K^+^ currents). This set of membrane ionic currents constitute the basis of the electrical excitability of neurons. The Na^+^ current form the upstroke of the action potential and the ensuing K^+^ currents repolarize the membrane (Figure 2A,C,E). The inward and outward current density of the dorsal root ganglion (DRG) neurons of Wistar rats cultured in the control substrate and TiO_2_ thin films are evaluated. The current density is obtained dividing the current amplitude with respect to the membrane capacitance. The inward and outward current are measured at −20 and +40 mV, respectively.

**Figure 3:**
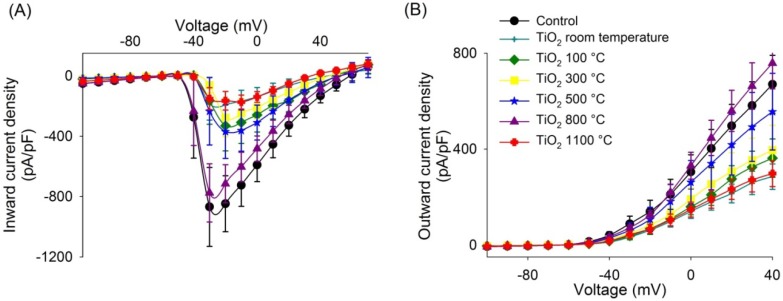
Inward and outward current density of the DRG neurons cultured in control substrate and TiO_2_ thin films. (**A**) Average inward current density as a function of voltage; (**B**) Average outward current density as a function of voltage.

The inward current density is significantly less for DRG neurons grown on the TiO_2_ thin film surfaces prepared at room temperature and at 300 and 1100 °C than that for DRG neurons grown on the control substrate (Table 2 and Figure 3A). Regarding other TiO_2_ thin films annealed at temperatures of 100, 500 and 800 °C, there is no significant difference compared with control substrate (Table 2 and Figure 3A). The amplitude of the outward current density of the DGR neurons cultured in the TiO_2_ thin films surfaces at room temperature and 1100 °C are lower than those obtained for neurons cultured on the control substrate (Table 2 and Figure 3B). The outward current density in other TiO_2_ thin films is not significantly different with respect those measured of the control substrate (Table 2 and Figure 3B).

In current clamp experiments, the membrane voltage of the cells is about −60 mV and under depolarizing current pulse injection, the DRG neurons are capable of firing typical action potentials of similar morphology both in control substrate and TiO_2_ films surfaces (Figure 2B,D,F).

**Figure 4:**
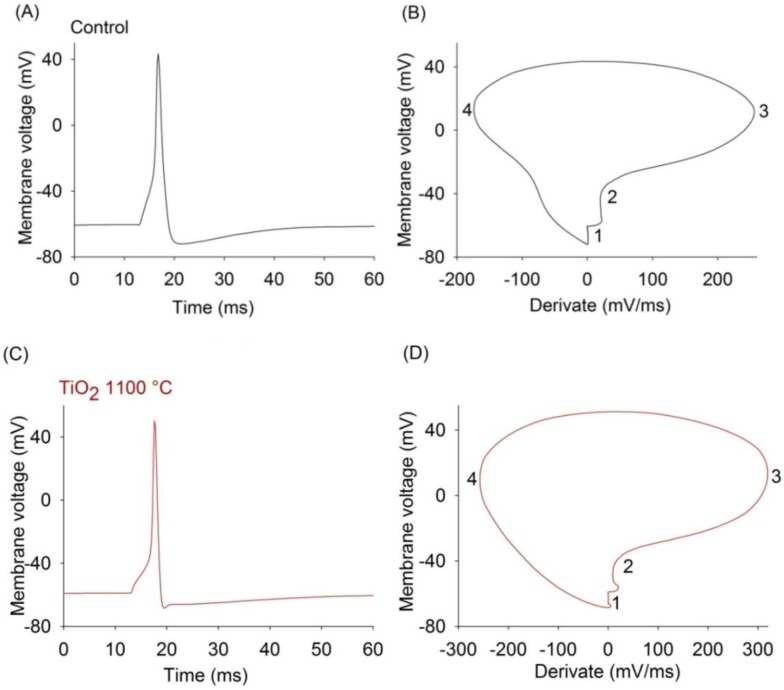
Representative action potential recordings of the DRG neurons on control substrate and TiO_2_ thin films annealed at 1100 °C. (**A**) Action potential produced by a 100 pA depolarizing current pulse; (**B**) Phase-plane plot of the action potential shown in panel A. 1—AHP, 2—threshold, 3—maximum depolarization rate, 4—maximum repolarization rate; (**C**) Action potential caused by 100 pA depolarizing current pulse in a neuron cultured on the TiO_2_ film; (**D**) Phase-plane plot for the action potential shown in panel C. 1—AHP, 2—threshold, 3—maximum depolarization rate, 4—maximum repolarization rate.

To analyze the properties of action potentials, we recorded the response to current pulses of DRG neurons grown on the control substrate and TiO_2_ thin films surfaces (Figure 4). The most of the action potential parameters measured of the TiO_2_ films surfaces are not significantly different from those measured in the control substrate (Table 3). Only the action potential depolarization rate measured of the TiO_2_ film surface annealed at 500 °C (*n* = 6) is significantly lower than that of the control substrate (Table 3; *p* = 0.039). In addition, the threshold of the TiO_2_ films surfaces annealed at 100 and 500 °C are significantly lower than that of the control substrate (*n* = 5, *p* = 0.013 and *n* = 6, *p* = 0.017 respectively).

**Table 2:** Inward and outward current density in DRG neurons cultured in the control substrate and TiO_2_ thin films surfaces prepared at room temperature and annealed at temperatures between 100 and 1100 °C.

Current Density	Control (*n* = 6)	TiO_2_ Room Temperature (*n* = 7)	TiO_2_ 100 °C (*n* = 5)	TiO_2_ 300 °C (*n* = 6)	TiO_2_ 500 °C (*n* = 5)	TiO_2_ 800 °C (*n* = 5)	TiO_2_ 1100 °C (*n* = 9)
pA/pF inward current	−813 ± 195	−218 ± 98 * *p* = 0.016	−462 ± 208 *p* = 0.250	−276 ± 126* *p* = 0.043	−375 ± 178 *p* = 0.138	−715 ± 126 *p* = 0.695	−164 ± 62 * *p* = 0.003
pA/pF outward current	670 ± 121	285 ± 53 * *p* = 0.011	362 ± 79 *p* = 0.073	396 ± 103 *p* = 0.115	555 ± 103 *p* = 0.576	758 ± 107 *p* = 0.606	298 ± 54 * *p* = 0.012

Mean ± standard error, * *p* < 0.05.

**Table 3:** Properties of action potentials of DRG neurons grown on control substrate and TiO_2_ thin film.

Action Potential Parameters	Control (*n* = 7)	TiO_2_ Room Temperature (*n* = 5)	TiO_2_ 100 °C (*n* = 5)	TiO_2_ 300 °C (*n* = 6)	TiO_2_ 500 °C (*n* = 6)	TiO_2_ 800 °C (*n* = 6)	TiO_2_ 1100 °C (*n* = 5)
Resting membrane potential	−60 ± 0.5	−62 ± 2	−62 ± 1	−60 ± 0.4	−61 ± 2	−60 ± 0.2	−60 ± 1
Amplitude of the action potential (mV)	109 ± 7	106 ± 8	113 ± 8	109 ± 5	104 ± 3	108 ± 9	95 ± 10
Duration 50% (ms)	1.2 ± 0.3	1.3 ± 0.3	0.81 ± 0.1	1.2 ± 0.2	2.3 ± 0.6	0.84 ± 0.1	1.1 ± 0.3
Maximum depolarization rate (mV/ms)	318 ± 42	236 ± 53	334 ± 59	253 ± 50	181 ± 39 *	311 ± 62	209 ± 66
Maximum repolarization rate (mV/ms)	−118 ± 23	−94 ± 17	−170 ± 17	−145 ± 21	−91 ± 37	−164 ± 23	−155 ± 40
Threshold (mV)	−36 ± 2	−34 ± 4	−24 ± 4 *	−30 ± 4	−23 ± 4 *	−34 ± 2	−32 ± 4
Amplitude of the AHP (mV)	−10 ± 2	−6 ± 2	−8.5 ± 1	−9 ± 1	−9 ± 2	−9 ± 0.4	−10 ±0.5

Mean ± standard error, * *p* < 0.05.

In the control glass substrate and TiO_2_ films annealed at room temperature and 100 °C, the 37% of the DRG neurons produced repetitive firing when were subjected to depolarizing current pulse injection. In the other TiO_2_ films only 33% of the DRG neurons produced two or more action potential.

## 3. Experimental Section

### 3.1. TiO_2_ Films Deposition

**Figure 5:**
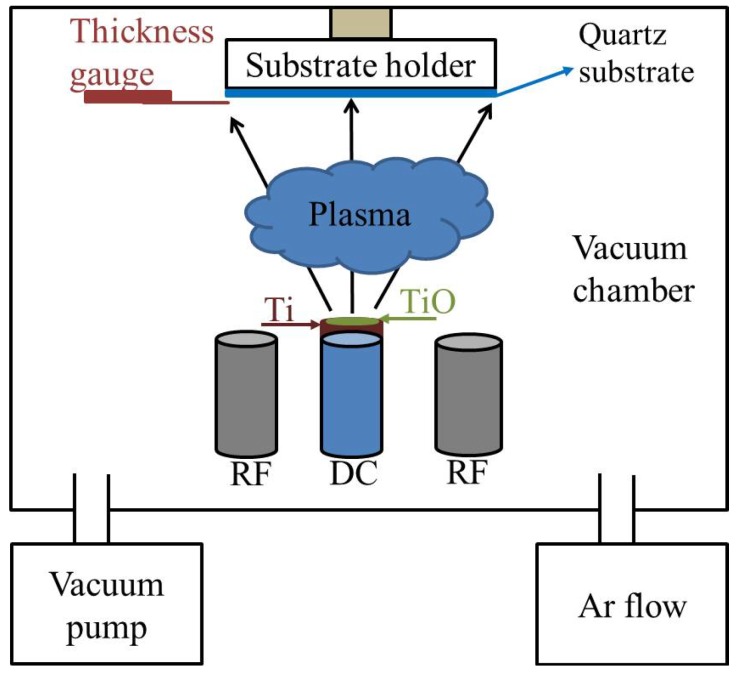
The parameters used in the deposition of TiO_2_ thin films were: pressure 5.6 × 10^−6^ Torr, Ar flow 15 sccm, time 60 min, power 100 W and substrate temperature 25 °C.

TiO_2_ thin films are deposited on a quartz substrate at room temperature by DC magnetron sputtering using a titanium target, which has a diameter of 50.8 mm. A TiO_2_ ceramic material is located on a 20% of the titanium target surface, in which both materials registered a purity of 99.99%, respectively. First, the quartz substrate is cleaned using ultrasonic baths of C_3_H_6_O, C_2_H_6_O, and distilled water during 5 min at room temperature. This procedure is repeated four times and, after, the TiO_2_ thin films depositions are made under an Argon (Ar) atmosphere and a chamber pressure of 5.6 × 10^−6^ Torr. The Ar flow is kept to 15 standard cubic centimeters per minute (sccm) during the TiO_2_ films deposition by DC magnetron sputtering. The DC power and substrate temperature are controlled to 100 W and 25 °C, respectively. Figure 5 shows a reactive DC magnetron-sputtering system, which is used to deposit TiO_2_ thin films on a quartz substrate (25.4 mm × 25.4 mm). Next, TiO_2_ films are subjected to thermal-annealing treatment to achieve their phase transformation. For this treatment, a thermo scientific thermolyne muffle furnace (model F48025-60-80, Thermo Fisher Scientific Inc., Waltham, MA, USA) is used to keep the temperature during one hour in each heat treatment. The time of each heat treatment is lower than that reported in elsewhere [40]. In addition, it is enough to reach the required recrystallization and transformation phases. Finally, TiO_2_ thin films are annealed at different temperatures (100, 300, 500, 800 and 1100 °C) to the anatase-to-rutile phase transformation.

### 3.2. Cell Culture

Experimental procedures involving animals are reviewed and approved by the Institutional Animal Care and Use Committee (CICUAL) of Vice-rectory for Research and Graduate Studies of Autonomous University of Puebla (VIEP-BUAP). Animal care and experimental procedures are performed in accordance with Mexican national regulations on Animal Care and Experimentation (NOM-062-ZOO-1999) [41]. These regulations are made to minimize the animal suffering and to reduce the number of used animals, as outlined in the Guide to the Care and Use of Laboratory Animals of the National Academy of Sciences (NAS). For the cell culture, Wistar rats (P7-P10) are euthanized by decapitation and their dorsal root ganglia are isolated from the vertebral column. Next, the tissues of the dorsal root ganglia are incubated in culture medium L-15 (Gibco, Thermo Fisher Scientific Inc., Waltham, MA, USA) added with 0.125% collagenase and 0.125% trypsin during 30 min at 37 °C. The dorsal root ganglia are subsequently washed with L-15 medium, and dissociated by mechanical agitation. In addition, dissociated cells are placed on both a control substrate (glass without TiO_2_ films) and TiO_2_ thin films, respectively, during a necessary time to allow the adherence of the cells with the TiO_2_ surface. Later, fetal bovine serum (FBS) 10% (Gibco) and L-15 medium (modified for CO_2_ by supplementing with 10 mM NaHCO_3_ and 10 mM 4-(2-hydroxyethyl)-1-piperazineethanesulfonic acid (HEPES; Sigma-Aldrich, St. Louis, MO, USA), are added and this medium is complemented with100 IU/mL penicillin (Merck, Merck KGaA, Darmstadt, Germany). The cells are incubated at 37 °C under an atmosphere of 95% air-5% CO_2_ during 18 to 24 h until electrophysiological recording. 

### 3.3. Electrophysiological Recording and Data Analysis

Neurons of the dorsal root ganglia are identified under a phase contrast microscope (Nikon TMS, Tokyo, Japan) and classical patch clamp methods are used for electrophysiological recordings [42]. Patch clamp pipettes are pulled from borosilicate glass capillaries using a Flaming-Brown (80/PC; Sutter Instruments, San Rafael, CA, USA). Commonly, these pipettes had a resistance of 1 to 3.5 MΩ and the seal resistance to cell membrane exceeded 1 GΩ. We used a voltage clamp amplifier Axopatch 200B (Molecular Devices, Sunnyvale, CA, USA). Command signals and data acquisition are generated by an analog-digital converter (Digidata 1200, Molecular Devices), which is controlled by the pClamp 10 software (Molecular Devices) [43]. Both voltage and current clamp experiments are performed. In these experiments, cell capacitance and series resistance are electronically compensated (80%) when the whole-cell configuration is obtained. Electrophysiological signals are low-pass-filtered at 5 or 2 kHz and digitized at 20 or 10 kHz. Cells are bathed with standard external and internal solutions, as shown in Table 4. In addition, internal solutions contain both 2 mM ATP-Mg and 1 mM GTP-Na. The pH levels of the external and internal solutions are adjusted at 7.4 with NaOH and 7.2 with KOH, respectively. Osmolarity is monitored by a vapor pressure osmometer (Wescor, Logan, UT, USA), which is adjusted with dextrose at 300 mOsm and 310 mOsm for internal and external solutions, respectively.

**Table 4:** Composition of standard solutions (in mM).

Solution	NaCl	KCl	CaCl_2_	MgCl_2_	HEPES	EGTA *
External	140	5.4	1.8	1.2	10	–
Internal	10	135	0.134	5	5	10

EGTA *—ethylene glycol tetraacetic acid.

For voltage clamp recordings, the cells are held at a holding potential of −60 mV (resting membrane potential), in where membrane currents are caused by voltage variation. The voltage shift is obtained using 10 mV voltage steps from −100 to 50 mV with duration of 800 ms, followed by a constant voltage of 20 mV during 200 ms. Thus, total membrane ionic current is recorded and the inward and outward currents are gave rise to a cell excitability and action potential generation.

For the current clamp experiments, the filters are open to 10 kHz and the pClamp software [43] generated square current pulses from −0.5 to 0.5 nA with 0.1 nA steps and 100 ms. The characteristics of the action potentials, caused by 1 ms suprathreshold pulses, are analyzed off-line using Clampfit (Molecular Devices) and Origin 8.0 (Microcal Software, Northampton, MA, USA). The phase-plane graphic of the cell voltage is obtained by plotting the first derivative of the membrane voltage (*V*) with respect to time (*t*) *versus* the membrane voltage [44]. The threshold of the action potential is the voltage at which whose d*V*/d*t* increases suddenly, and action potential amplitude is the voltage difference between the maximum peak and resting potential. The duration of the action potential at 50% is defined as the duration of the action potential measured at the half of its amplitude. The maximum depolarization and repolarization rate are obtained from the phase-plane graphic as the maximum and minimum d*V*/d*t* values, respectively. The after-hyperpolarization (AHP) is defined as the potential difference between the minimum voltage value after the action potential and the membrane potential preceding the action potential. All results are reported as the mean ± standard error. For all comparisons between the control glass and TiO_2_ thin film surfaces a Student’s *t*-test was used, considering as significant a *p* < 0.05.

## 4. Conclusions

TiO_2_ thin films deposited by magnetron sputtering at room temperature and annealed with different temperatures (100, 300, 500, 800 and 1100 °C) were studied, analyzing their morphology and biocompatibility. The XRD patterns showed the transition of the anatase phase to rutile phase. To evaluate the biocompatibility *in vitro* of the TiO_2_ thin films, primary cultures of dorsal root ganglia (DRG) neurons were performed, evaluating their survival rate and electrophysiological activity. As a biological system, DRG neurons were selected because their normal functional electrical activity in the materials can be parametrically defined, which is a clear indication of biocompatibility. The electrophysiological recordings showed that the DRG neurons can be maintained in culture during 24 h on TiO_2_ thin films surfaces. The electrophysiological properties of the neurons, indicated the cells viability and showed that they are functionally similar to those deposited on the control substrate. In the TiO_2_ thin films at 800 °C, the currents amplitudes were similar to those measured on control substrate. At this temperature, the TiO_2_ thin films has morphological characteristics that could create an environment adequate for the cells due to the fact that they presented a normal set of membrane ionic channels. The result of these experiments confirmed the feasibility of using TiO_2_ thin films as a biocompatible material for the stimulation and recording of electrical activity of DRG neurons in culture.

Future work will include the deposition of TiO_2_ thin films on the microelectrodes surface and the readout circuit to fabricate CMOS-MEMS devices. This circuit will allow recording of other parameters of biological relevance such as micropotentials due to pH.
